# Cross-Cultural Differences in the Generation of Novel Ideas in Middle Childhood

**DOI:** 10.3389/fpsyg.2020.01829

**Published:** 2020-08-13

**Authors:** Moritz Köster, Relindis Yovsi, Joscha Kärtner

**Affiliations:** ^1^ Institute of Psychology, Free University Berlin, Berlin, Germany; ^2^ Independent Consultant, Brussels, Belgium; ^3^ Department of Psychology, University of Münster, Münster, Germany

**Keywords:** cross-cultural comparison, creativity and innovation, cognitive development, alternative uses task, middle childhood

## Abstract

Innovation and creativity have recently been in the center of the debate on human cultural evolution. Yet, we know very little about childrens’ developing capacity to generate novel ideas, as a key component of innovation and creativity, in different cultural contexts. Here, we assessed 8‐ to 9-year-old children from an autonomous and a relational cultural context, namely Münster (urban Germany; *n* = 29) and Banten (rural Cameroon; *n* = 29). These cultural contexts vary largely in their ecology, social structure, and educational system, as well as the cultural models on children’s individual development and thinking. Therefore, they provide an optimal contrast to investigate cultural similarities and differences in development of creative capacities. We applied classical divergent thinking tasks, namely an alternative uses task and a pattern association task. In these tasks, children are asked to generate as many ideas as possible what an object could be used for or what a pattern could be. First, our study revealed a good internal consistency and inter-task correlations for the assessment of children’s fluency and the generation of unique ideas in both cultures. Second, and most critically, we found significantly higher levels of creative capacities in children from Münster in contrast to Banten. This was reflected in both a higher number of ideas (fluency) and a higher number of unique ideas (uniqueness). Third, looking at the type of answers that children gave in the alternative uses task, we found that children from Münster and Banten uttered a similar number of conventional ideas, but that children from Münster uttered more ideas to manipulate an object, invent novel things with an object, and involve an object in play or pretend play, or in a fantasy story. This demonstrates that early creative development is strongly influenced by the cultural context and substantiates the cultural nature of human cognitive development.

## Introduction

In the current debate on human unique cognitive capacities, a central role has been ascribed to both innovation and imitation as two central psychological mechanisms underlying human cultural evolution (e.g., Legare and Nielsen, 2015; van Schaik, 2016). According to these accounts, high-fidelity imitation is key to acquire the cultural repertoire, and this competence emerges early in development. Innovation is equally important and complementary in the sense that it allows refining and expanding the cultural repertoire within and across generations. Ontogenetically, innovation emerges later, but imitative capacities remain relevant throughout development. Increasing competence and experience of the individual allows for the development of higher levels of innovation, with adolescents and young adults being the most likely innovators (van Schaik, 2016). Thus, individual problem solving and creativity play a central role as the driving forces of innovation in human cultures. In support of this idea, Neldner et al. (2019) report data that show that, across three different tasks and five different cultures, children are relatively poor tool innovators before age five and become much more proficient by age nine, across cultures.

At the same time, Neldner et al. (2019) found support for cross-cultural variation in children’s proclivities to innovate. More specifically, while innovation across the non-Westernized small-scale society groups was, in general, similar, the innovation of children from a Westernized city was considerably higher. In a study on imitative flexibility in 6‐ to 8-year-olds from a US-American metropole and a small-scale society in Ni-Vanuatu, Clegg and Legare (2016) found Ni-Vanuatu children engaged in higher imitative fidelity than US-American children. As one reason, the authors discuss that caregivers in US-American educated urban middle-class families favor divergent thinking in young children, rather than conformity, which might support children’s individual inventiveness, leading to these cultural differences. This interpretation is further supported by a study on the role of conformity in US-American educated urban middle-class and rural Ni-Vanuatu adults’ judgments of children’s intelligence (Clegg et al., 2017). Based on multivocal ethnography, this study found that US-American adults were less likely to endorse high-conformity children as intelligent, often citing creativity as a justification for their judgments. In contrast, Ni-Vanuatu adults were more likely to endorse Ni-Vanuatu high-conformity children as intelligent.

A central component of creativity and innovation is children’s capacity to generate novel ideas (i.e., divergent thinking) as an indicator of their creative capacities (Runco and Acar, 2012). That is, the generation of novel, original ideas (e.g., Guilford, 1950) is a critical prerequisite for the production, implementation, and dissemination of innovative and useful ideas and products (Puccio and Cabra, 2010; Sawyer, 2012). Specifically, divergent thinking tasks provide a measure for the quantity and originality of ideas that children generate (e.g., Wallach and Kogan, 1965; Guilford, 1966; Torrance and Ball, 1984). For example, in the so-called alternative uses tasks, children are asked to generate different ideas about the use of objects, or in a pattern association task, to generate several ideas on what a black-and-white pattern could be (Wallach and Kogan, 1965; Ward, 1968). Across several studies, these tasks have been found to be highly suited to assess children’s creativity, because they show a high reliability (i.e., a high internal consistency and inter-task correlations) and are clearly distinct from classical IQ measures (e.g., Wallach and Kogan, 1965; Pankove and Kogan, 1968; Cropley and Maslany, 1969). Furthermore, they have been applied across a broad age range (e.g., Ward, 1968) and have been shown to be relatively stable over time (Kogan and Pankove, 1972). Finally, especially the alternative uses and pattern association tasks seem to be suited for an application in non-Western and rural contexts because they are conducted with very simple materials, which are likewise familiar (objects) or novel (pattern) to children in these contexts (cf. Jurkat et al., 2020).

To date, a few studies have applied this or similar tasks in non-Western contexts, mostly looking at creativity in children from the Asian continent (e.g., Rudowicz et al., 1995; Rudowicz, 2003, 2004; Marsh, 2010), with a similar level of industrialization and education like in Western cultural contexts. Thus, in the light of the current debate on cross-cultural differences in imitation and innovation between Western and non-Western rural contexts of developing countries, outlined above, it would be intriguing to investigate cross-cultural similarities and differences in the generation of novel ideas during middle childhood in children from more diverse cultural contexts.

Toward this end, in the present study we selected two often-studied prototypical cultural contexts, which differ profoundly in their ecology, social structure, and educational system, namely the city of Münster with families from the educated middle-class in Germany, and the village of Banten, a subsistence-based farming ecology in rural Cameroon, near the municipal of Kumbu. The city of Münster is a typical Western context, more specifically representing a prototype of an independent (Markus and Kitayama, 1991) – or autonomous (Keller and Kärtner, 2013) – cultural context. Families and household sizes are usually small. Parents are occupied in professional jobs and have high levels of formal education, and children usually attend the kindergarten from age two or three and visit the school from around age six. Parental behavior and socialization focus on autonomy and individual development, such as making choices independently (Kärtner, 2015; Köster et al., 2016). Children from the Nso culture in the village of Banten, a typical non-Western context, grow up in large, extended family settings in subsistence-based villages. This cultural context has been characterized as relational (Keller and Kärtner, 2013). This is, socialization practices focus on obedience and taking on responsibilities, which is associated with social roles in hierarchical social relationships (Keller, 2007). Most parents are farmers and engage their children in household tasks and fieldwork from early on (Köster et al., 2018), and do so in an assertive and demanding tone of voice (Köster et al., 2016). Children visit the preschool from around age four and the educational style is dominated by a strong hierarchal relation between pupils and the teacher.

Children from both cultural contexts participated in child-friendly versions of the alternative uses and a pattern association task (adapted from Ward, 1968), to assess their abilities for divergent thinking. We assessed children’s generation of novel ideas, by the number of ideas (fluency) and the number of unique ideas (uniqueness), as two classical indicators for divergent thinking. Our main proposal was that children from urban Germany would be more fluent in their generation of novel ideas and that they would generate more unique ideas. In addition, to get a better idea on the cross-cultural similarities and differences concerning the content of the ideas generated, we rated the different types of object uses in the alternative uses task (conventional uses, object manipulations, innovative ideas, play ideas, pretend play suggestions, and fantasy ideas). Our hypothesis was that children from urban Germany may generate more innovative, play, pretend play, and fantasy ideas, as an expression of their higher levels in the generation of novel and creative ideas.

## Materials and Methods

### Participants

The final sample consisted of 29 8‐ to 9-year-old children from urban Germany (*M* = 8.69 years, *SD* = 0.59, 52% girls) and 29 same-aged children from rural Cameroon (*M* = 8.25 years, *SD* = 0.69, 69% girls). One additional child in Münster came to the lab but did not want to participate. All other children could be included in the analysis.

In Münster, families were contacted *via* a database from the university. In Kumbo, children were recruited in cooperation with local schools. Families received financial compensation in Kumbo and cinema coupons in Münster for their participation. Note that in Kumbo, the date of birth was not exactly known for most children and thus estimated by the mothers. Informed written consent was obtained from parents in both contexts, and children gave informed assent.

### Stimuli and Procedure

Children took part in one experimental session. In Kumbo, the laboratory was set up in a quiet room of the school, whereas in Münster, participants visited the laboratory of the university with a parent. During the session, the child and the experimenter were alone in the room with two chairs and a table and the room was kept as plain as possible. This is, we removed all loose objects in the room (e.g., pictures, rubbish bin, etc.) to avoid that these objects could support children in forming specific ideas.

Children first took part in an alternative uses task and then in a pattern association task. The procedure of both tasks was adapted, as close as possible, from a study with children at the same age, by Ward (1968). Both tasks were introduced as a game. All sessions were video-recorded for subsequent transcription and coding of the ideas of the child.

Note that these tasks are commonly differentiated as being semantical (alternative uses task) and figurative (pattern association task) within the Torrance Tests of Creative Thinking (TTCT; Torrance, 1984). Furthermore, there are more recently developed tests in the field, such as the Creative Thinking-Drawing Production (TCT-DP), which may be better in reflecting the processes of creative thinking. However, in the present study we selected those two tasks, because our main focus was on children’s generation of novel ideas, these tasks were probed with children at school-age (Ward, 1968), and we considered them highly suitable (in terms of comparability) for an application the two highly different cultural contexts.

Note that we kept the order of tasks constant across cultures, because the main focus was on the difference between cultures and not the differences between tasks. We started with the alternative uses task, because it is closer to children’s daily experiences, such that we considered it the better start for children to warm up and understand the structure of the tasks.

#### Alternative Uses Task

**Figure 1:**
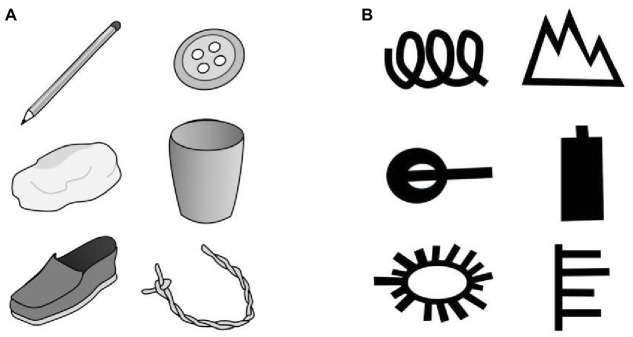
The objects and pattern used in the creativity tasks. **(A)** Schematic drawings of the objects of the alternative uses task. The objects given to the children were real objects. **(B)** The pattern presented to the children in the pattern association ask. Children were presented the pattern printed on a card, one after another. Note that the item on the upper left corresponds to example item, used for the instruction and training of each task.

The task was conducted for five real objects, one after another: a piece of string, a cup, a shoe, a stone, and a button (see Figure 1A). The task started with a training object (a pencil, not included in the final analysis) and was introduced as a game called *What can you use it for?* The experimenter began by introducing the first object “We are first going to play with a pencil [handing over a pencil to the child]. Now, I want you to tell me all the things you can think of that you can do with a pencil, or what you can play with it or what you can make with it.” The experimenter positively acknowledged each idea (e.g., “yes, this is a good idea”) and encouraged the child to continue (e.g., “What else can you think of? What else can you do, play or make with a pencil?”). Children could continue until they stated that they had no further ideas. In case the child generated less than four ideas, the experimenter encouraged the child one more time to think of further uses. For the first object (i.e., the pencil), the experimenter suggested two additional uses in the end. This was to be sure that children would understand the task correctly (i.e., “You could also use it to dig in the dirt, you could use it as a flagpole and put a small flag on it, or you could put wheels on it to create a toy car”). Thereafter, the experimenter repeated the same protocol for the five test objects.

#### Pattern Association Task

The task was conducted with five abstract patterns (see Figure 1B), each printed on a card. Again, the task was introduced by the experimenter with a training pattern (Figure 1B, upper left pattern), who said “Now we play a game called *What could this be?* The first thing we play with is this pattern [showing the pattern to the child]. Now, I want you to tell me all the things you can think of that this could be.” The behavior of the experimenter was identical as for the alternative uses task (see above). Again, for the first pattern the experimenter added some more suggestions in the end (i.e., “Look, this could also be a tail of a pig, three times the letter ‘e’ or a fence”).

### Data Coding and Analysis

#### Data Preprocessing

All ideas uttered by the child were transcribed from video. Children in Cameroon spoke Lamnso, the local language, and their ideas were transcribed into English by a local research assistant.

In the first step, we excluded ideas that were redundant, irrelevant, or too unspecific. An idea was redundant, if it was uttered before by the child for a specific object, or if it implied the same action performed with the object, or fulfilled exactly the same function as an idea that was uttered before. Examples for redundant object uses would be that a pencil could be used for painting or drawing, or that a cup could be used as a container for stones or as a container for earth. For the pattern, ideas would be redundant if they were repeated, highly similar, or synonymous. Examples for redundant ideas would be house or villa, or a spoon or a wooden spoon. An idea was excluded as irrelevant in case it was not a response to the question (e.g., this is black and white) or if the uttered idea could not be related to the object (e.g., to use a pencil for a papernose). In accordance with Ward (1968), an idea could not be discarded as irrelevant, if it was uttered by at least two children. Finally, we excluded an idea as too unspecific, in case it did not imply a specific action or purpose. Examples for unspecific ideas would be one could “put the object somewhere” or that one could “make something out of it.”

Finally, before the analysis, we identified ideas across participants, which referred to the same use or purpose. Those ideas received the same label and were considered as identical ideas. Examples for ideas considered as identical were to tear something apart or to pull something apart or also the examples given above for ideas that were considered redundant to each other. The preprocessed data were used in all subsequent analysis steps.

#### Fluency and Uniqueness

In a first step, we analyzed participants’ fluency, that is, the mean number of different ideas that a participant generated for each object. Furthermore, we analyzed the number of unique ideas, the mean number of ideas per object that was only uttered by one child.

#### Types of Object Uses

To better understand which aspects the ideas of the children between cultures would be similar or different, we developed six different categories of ideas (for a similar approach, see Oncu et al., 2015). These categories were Conventional, Manipulation, Innovation, Play, Pretend Play, and Fantasy.

##### Conventional

Conventional means that the idea corresponds to the intended purpose of an item, namely that its use is functional with a reasonable (not playful) goal. Because the number of conventional uses is defined by an object, we developed a list with the conventional uses for each object. For example, for a pen, this was to write, paint, draw, or to sharpen it (see also Oncu et al., 2015).

##### Manipulation

Manipulation referred to a non-functional change of the condition or location of an object that does not have a playful character. For example, to break the pencil apart or to throw it into a corner.

##### Innovation (and Tool Use)

Innovation and tool use were defined as novel uses that are realistic, functional, and follow a reasonable goal but are not a conventional use of this object. According to Beck (1980), this would include the creation of new tools, and specifically involve four distinct actions: detach, subtract, reshape, and add/combine the object (see also Neldner et al., 2019, for a cross-cultural study on tool innovation). An example would be to use the pen as a flagpole.

##### Play

Play was defined as any action with the object that does not follow an instrumental goal. Examples would be to throw a pen up and catch it again or to balance it on a finger.

##### Pretend Play

Pretend Play was defined as any symbolic use of the object. Namely, any use where the identity of the object was alternated to replace another object (Bruner, 1972; see also Oncu et al., 2015). An example would be that a pen would be used as a telephone.

##### Fantasy

Fantasy ideas were those that were unrealistic, in the sense of a fairytale character. For example, beyond the symbolic character of pretend play, the object was turned into something that does not exist or used for something that is not possible. An example would be that the child would stand on the pencil and fly with it or as a magic sword to fight dragons.

We established the interrater reliability for the coding of the categories for >20% of the data (i.e., six children in each context). The agreement between two independent coders was good (Münster: Cohens *κ* = 0.85, Banten: Cohens *κ* = 0.81).

## Results

### Fluency and Uniqueness

First, we tested the internal consistency for the fluency and uniqueness scores across objects and patterns. This revealed high consistencies for both cultures and tasks (see Table 1). Furthermore, we tested the correlations between the four scores, separated for both cultures. This revealed highly significant correlations between the different scores within cultures (see Table 2). Thus, in accordance with previous studies with children, the tasks seem to be reliable in assessing children’s production of creative ideas and did so in both cultural contexts.

**Table 1:** Consistency of fluency and unique responses across the five test items.

	Object		Pattern	
	Fluency	Uniqueness	Fluency	Uniqueness
Münster (urban Germany)	0.97	0.92	0.89	0.85
Banten (rural Cameroon)	0.92	0.78	0.89	0.83

Values indicate Cronbach’s *ɑ*.

**Table 2:** Correlations between the different scores.

	2.	3.	4.
Münster (urban Germany)			
Object fluency	0.93^***^	0.47^**^	0.52^**^
Object uniqueness		0.36^(*)^	0.44^*^
Pattern fluency			0.97^***^
Pattern uniqueness			
Banten (rural Cameroon)			
Object fluency	0.88^***^	0.69^***^	0.73^***^
Object uniqueness		0.54^**^	0.56^**^
Pattern fluency			0.92^***^
Pattern uniqueness			

(*) *p* < 0.10;

* *p* < 0.05;

** *p* < 0.01;

*** *p* < 0.001.

**Figure 2:**
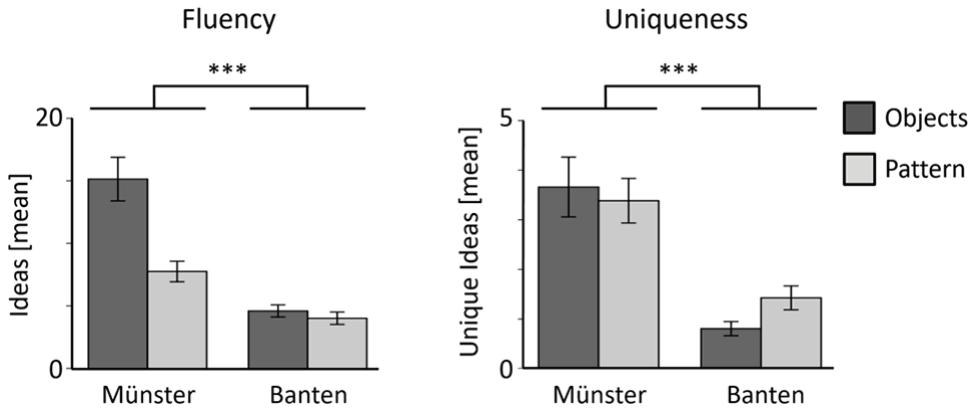
The mean fluency and unique ideas for children from both cultures. Fluency corresponds to the mean number of ideas uttered by the children, per object. Uniqueness corresponds to the mean number of ideas only uttered by one child, per object. ^***^ *p* < 0.001.

The mean values and SEs for children’s fluency and uniqueness are displayed in Figure 2. We tested the cross-cultural differences in fluency and uniqueness, by subjecting those scores to two separate mixed ANOVAs with Culture (Münster, Banten) as between-subject factor and Task (Object, Pattern) as a within-subject factor. Children’s fluency was much higher in Münster compared to Banten, main effect Culture, *F*(1, 56) = 35.60, *p* < 0.001, *ηp^2^* = 0.39, and much higher for Objects compared to Patterns, main effect Task, *F*(1, 56) = 23.74, *p* < 0.001, *ηp^2^* = 0.30. There was also a significant Culture × Task interaction, *F*(1, 56) = 17.29, *p* < 0.001, *ηp^2^* = 0.24, indexing that in Münster the difference in fluency between Objects and Pattern was higher than Banten. Children’s number of unique ideas were also much higher in Münster compared to Banten, main effect Culture, *F*(1, 56) = 25.59, *p* < 0.001, *ηp^2^* = 0.31. However, we did not find a main effect of Task, *F*(1, 56) = 0.31, *p* = 0.579, *ηp^2^* = 0.01, and also no interaction between Culture × Task, *F*(1, 56) = 2.11, *p* = 0.152, *ηp^2^* = 0.04.

**Figure 3:**
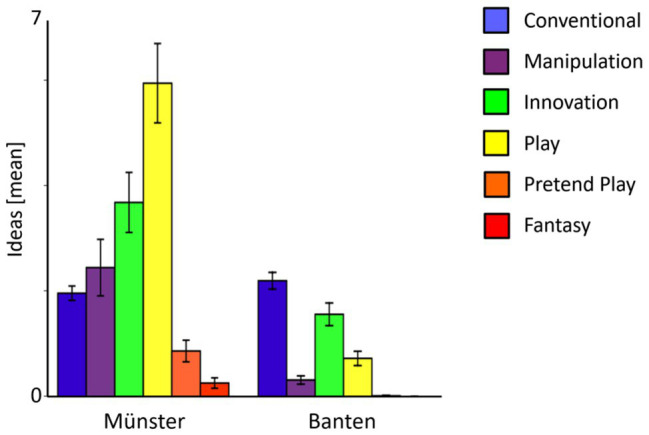
The mean number of object uses uttered within each of the six categories for the alternate uses task (Conventional, Manipulation, Innovation, Play, Pretend Play, and Fantasy). The cross-cultural comparison was not significant for Conventional responses (*p* = 0.456), but highly significant for all other categories, namely Manipulation (*p* < 0.001), Innovation (*p* = 0.001), Play (*p* < 0.001), Pretend Play (*p* < 0.001), and Fantasy (*p* = 0.012).

### Types of Object Uses

We tested the cross-cultural differences in the types of object use, by subjecting those scores to a mixed ANOVA with Culture (Münster, Banten) as between-subject factor and Type (Conventional, Manipulation, Innovation, Play, Pretend Play, and Fantasy) as within-subject factor, displayed in Figure 3. Looking at which categories children’s ideas differed, we found a main effect of Culture, *F*(1, 56) = 33.85, *p* < 0.001, *ηp^2^* = 0.38, reflecting the difference in fluency between both cultures. We further found a main effect Type, *F*(5, 280) = 38.87, *p* < 0.001, *ηp^2^* = 0.41, and a significant Culture × Type interaction, *F*(5, 280) = 24.31, *p* < 0.001, *ηp^2^* = 0.30. Looking at the cross-cultural comparison at the level of single types, the only type of object uses that did not differ significantly between cultures was the Conventional use, *t*(56) = −0.75, *p* = 0.456. Children from Münster gave significantly more responses in all other categories, namely Manipulation, *t*(56) = 3.96, *p* < 0.001, Innovation, *t*(56) = 3.60, *p* = 0.001, Play, *t*(56) = 6.87, *p* < 0.001, Pretend Play, *t*(56) = 4.14, *p* < 0.001, and Fantasy, *t*(56) = 2.59, *p* = 0.012.

## Discussion

Most critically, the present study revealed significantly higher levels in the generation of novel and original ideas in children from educated urban middle-class families in Münster, Germany, in contrast to children from families with a basic level of formal education that live in subsistence-based farming ecology in Banten, rural Cameroon. This was reflected in a higher fluency and uniqueness of ideas that children from Münster generated for objects and patterns. Furthermore, the type of answers that children gave in the alternative uses task showed that children from Münster and Banten uttered a similar number of conventional ideas, but that children from Münster uttered more ideas in all other categories. These were ideas to manipulate the object, invent novel things with the object, and involve the object in a play, a pretend play, or in a fantasy story.

Consistent with earlier studies (Wallach and Kogan, 1965; Ward, 1968), we found that the tasks were highly reliable (across different objects and patterns used) and that the different measures for children’s creative capacities (fluency for objects, uniqueness for objects, fluency for patterns, and uniqueness for patterns) were highly correlated. Although all tasks were framed as a game to motivate the children, it is difficult to disambiguate the degree to which the results in the tasks are influenced by children’s tendency to interact and converse with the experimenter or their verbal fluency. Importantly and in support of the conclusion drawn above, we found that children from Münster and Banten did not differ in the number of conventional ideas they uttered, indicating that there was no general difference in children’s readiness to participate in the task and express their ideas to the experimenter.

These data nicely complement the findings on children’s innovation proclivities by Neldner et al. (2019). Using tool manipulation during a problem-solving task as the key indicator of innovation, they similarly found that, first, children were reasonably proficient innovators by age nine and, second, that innovation of children from a Westernized city was considerably higher than of children from small-scale societies. Together, this converging evidence points toward considerable cross-cultural variation in key capacities for creativity and innovation during middle childhood between WEIRD cultures and small-scale cultural communities.

The present findings further emphasize that the development of creativity and innovation depends largely on culture-specific learning experiences. Thereby, they raise intriguing questions for different aspects of children’s culture-specific learning experiences that explain the cultural variability in creativity. Noteworthy, as a result of the choice of two very different cultural contexts, it is a limitation of the present study that those contexts differ in a high number of social (i.e., socialization goals and parenting strategies) and ecological factors (i.e., household structure, educational system, parental education, urbanization, and mode of subsistence). Thus, it remains a matter of debate and potential future investigation to better understand which factors are critical in shaping early creative capacities. In the following, we will speculate on a few processes that may underly the cross-cultural variation identified here.

A central role in culture-specific developmental pathways is ascribed to the early parent-child interaction (Keller, 2007; Keller and Kärtner 2013; for an example on childrens’ cognitive development, see Köster and Kärtner, 2018). Consistent with the idea that cross-cultural variation in children’s innovation might be driven by caregivers’ ethnotheories about creativity or compliance as key features of the talented child (Clegg and Legare, 2016; Clegg et al., 2017), we have specifically chosen the two cultural contexts in the present study to reflect the prototype of an autonomous versus a relational cultural context, which have been shown to differ profoundly in parental values (Keller, 2007). For instance, parents in autonomous cultural contexts typically value independent thinking, uttering one’s opinion, and generating novel ideas (Keller, 2007) which are also reflected in various aspects of their parenting behavior (Keller et al., 2004; Kärtner, 2015; Köster et al., 2016). On the other hand, parents from relational cultural contexts typically value conformity and the respect of hierarchical social relations (Keller, 2007). On the behavioral level, this is reflected in an assertive and insistent way parents instruct their children (Köster et al., 2016). These differences in cultural values and practices are likewise reflected in the educational system. While in urban Germany, there is an emphasis on individual thinking and discourse, in rural Cameroon, the emphasis lies on repeating correct responses instructed by the teacher. Besides a potential role of parental socialization and the differences in educational contexts, children’s ecological environment may play a central role in the development of creativity and innovation. The environment of children in Western, urbanized contexts is largely enriched by toys and tools of all sorts, providing them with a diversity of experiences that may facilitate their generation of ideas what function objects could provide.

While these theoretical considerations fit neatly with the stark contrast in creative development found in school-aged children in the present study, for future research, it is essential to empirically test the role of different experiences children make across cultures on their innovative and creative potential (cf. Köster and Kärtner, 2019). For example, one could specifically test the effect of different parenting strategies and styles or different educational systems on children’s creative development.

To conclude, our findings substantiate a profound impact of the cultural context on children’s creative development and highlight that human cognitive development can only be fully understood in the broader developmental and cultural context.

## Data Availability Statement

The data underlying the analyses is available in the supplementary material of this article.

## Ethics Statement

Ethical review and approval was not required for the study on human participants in accordance with the local legislation and institutional requirements. Written informed consent toparticipate in this study was provided by the participants’ legal guardian/next of kin.

## Author Contributions

MK conceptualized and designed the study, assessed and analyzed the data. JK and RY provided critical feedback on the study design and data analysis. MK and JK wrote the paper, RY provided critical revisions. All authors approved the submitted version of the article.

### Conflict of Interest

The authors declare that the research was conducted in the absence of any commercial or financial relationships that could be construed as a potential conflict of interest.
